# Supplementary material for: Apolipoprotein-CIII O-Glycosylation Is Associated with Micro- and Macrovascular Complications of Type 2 Diabetes
Source: Int J Mol Sci. 2024 May 14;25(10):5365. doi: 10.3390/ijms25105365 (PMC11121677; doi:10.3390/ijms25105365)
Supplement: Supplementary file 1 [file ijms-25-05365-s001.zip › supplemetary Figure.pdf]

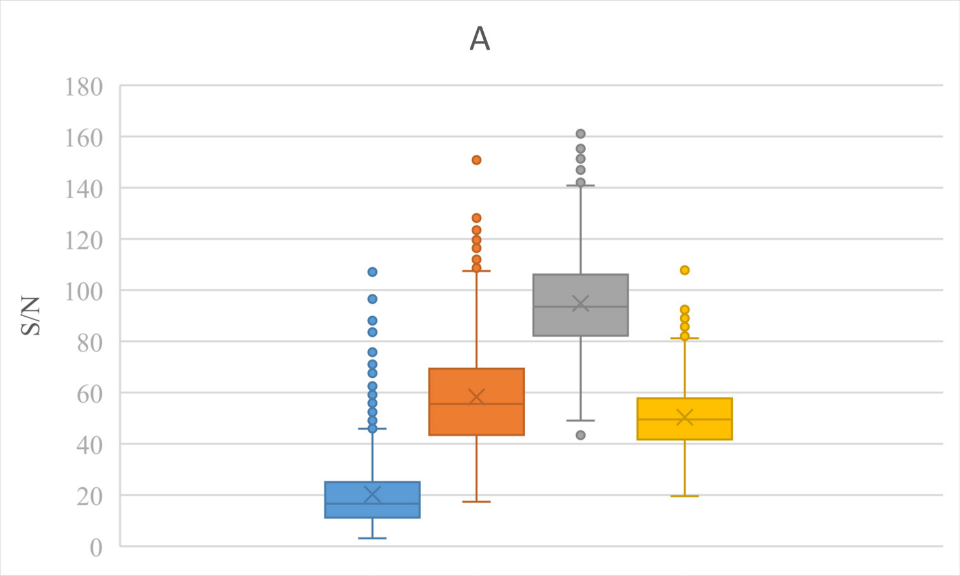
A
S/N
180
160
140
120
100
80
60
40
20
0

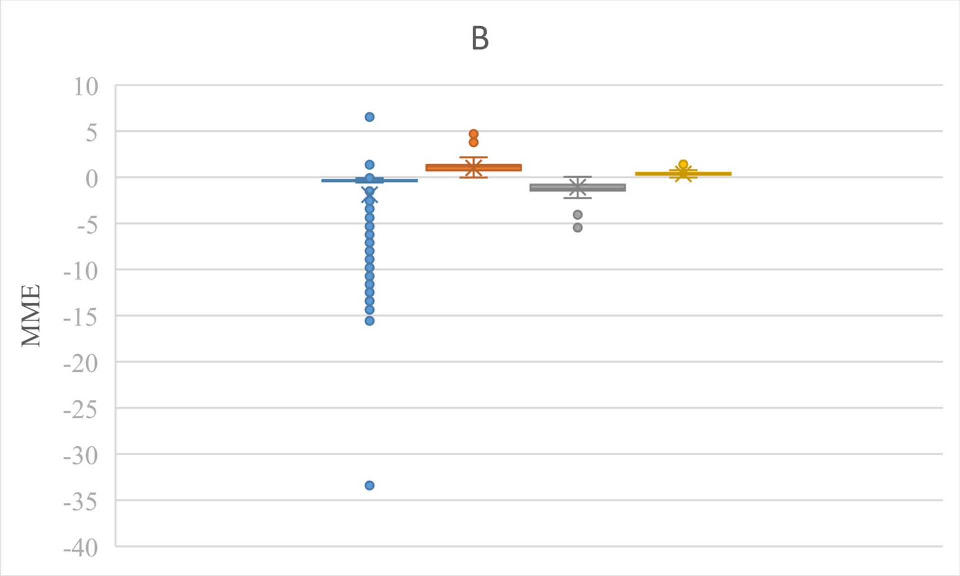
B
MME
10
5
0
-5
-10
-15
-20
-25
-30
-35
-40

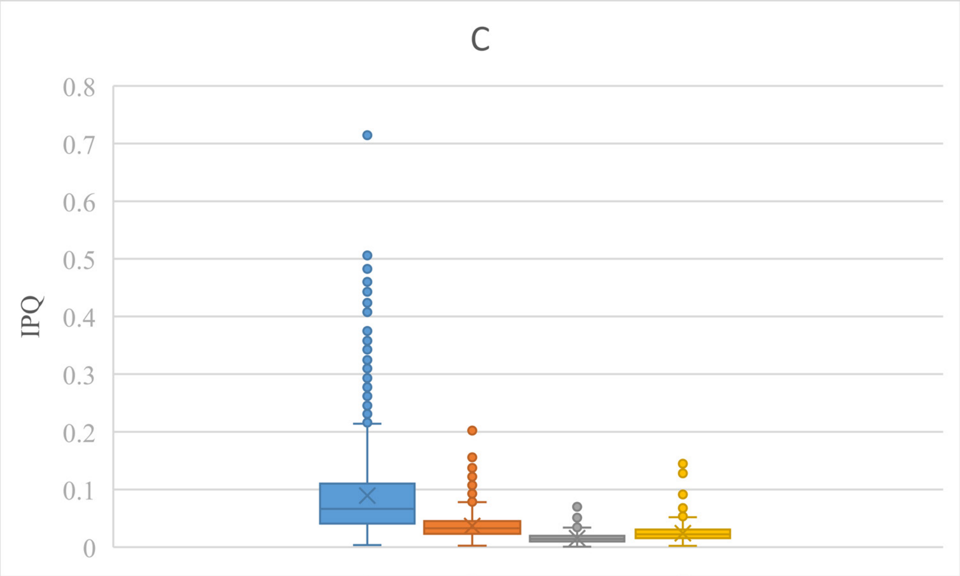
C
IPQ
0.8
0.7
0.6
0.5
0.4
0.3
0.2
0.1
0

Figure S1. Distributions of quality control parameters S/N (panel A), MME (panel B) and IPQ (panel C) for apo-CIII glycoforms in 1571 DiaGene samples.
S/N: signal-to-noise ratio; MME: mass measurement error; IPQ: isotopic quality pattern. Blue = apo-$CIII_{0a}$; orange = apo-$CIII_{0c}$; gray = apo-$CIII_1$; yellow = apo-$CIII_2$.
